# Emotionally clocked out: cell-type specific regulation of mood and anxiety by the circadian clock system in the brain

**DOI:** 10.3389/fnmol.2023.1188184

**Published:** 2023-06-27

**Authors:** T. Chase Francis, Alessandra Porcu

**Affiliations:** Department of Drug Discovery and Biomedical Sciences, College of Pharmacy, University of South Carolina, Columbia, SC, United States

**Keywords:** circadian rhythms, clock genes, plasticity, mesolimbic dopamine, serotonin, neuropeptides, SCN, hippocampus

## Abstract

Circadian rhythms are self-sustained oscillations of biological systems that allow an organism to anticipate periodic changes in the environment and optimally align feeding, sleep, wakefulness, and the physiological and biochemical processes that support them within the 24 h cycle. These rhythms are generated at a cellular level by a set of genes, known as clock genes, which code for proteins that inhibit their own transcription in a negative feedback loop and can be perturbed by stress, a risk factor for the development of mood and anxiety disorders. A role for circadian clocks in mood and anxiety has been suggested for decades on the basis of clinical observations, and the dysregulation of circadian rhythms is a prominent clinical feature of stress-related disorders. Despite our understanding of central clock structure and function, the effect of circadian dysregulation in different neuronal subtypes in the suprachiasmatic nucleus (SCN), the master pacemaker region, as well as other brain systems regulating mood, including mesolimbic and limbic circuits, is just beginning to be elucidated. In the brain, circadian clocks regulate neuronal physiological functions, including neuronal activity, synaptic plasticity, protein expression, and neurotransmitter release which in turn affect mood-related behaviors via cell-type specific mechanisms. Both animal and human studies have revealed an association between circadian misalignment and mood disorders and suggest that internal temporal desynchrony might be part of the etiology of psychiatric disorders. To date, little work has been conducted associating mood-related phenotypes to cell-specific effects of the circadian clock disruptions. In this review, we discuss existing literature on how clock-driven changes in specific neuronal cell types might disrupt phase relationships among cellular communication, leading to neuronal circuit dysfunction and changes in mood-related behavior. In addition, we examine cell-type specific circuitry underlying mood dysfunction and discuss how this circuitry could affect circadian clock. We provide a focus for future research in this area and a perspective on chronotherapies for mood and anxiety disorders.

## 1. Introduction

The increased importance of the biological circadian clock in health and disease has strongly contributed to the recent research trend investigating the role of circadian rhythms in the etiology and treatment of stress-related disorders including mood and anxiety disorders (Imamura and Takumi, 2022). Early evidence of circadian disruptions in mood disorders comes from clinical research. Diurnal variation in mood and disturbed sleep is commonly observed in major depressive disorder (MDD) and cyclic mood episodes and sleep variations are often clinical manifestations in bipolar disorder (McCarthy and Welsh, 2012). However, it is a two-way street: the causes of mood disorders such as acute or repeated stress and reward withdrawal can affect circadian rhythms and the alteration in circadian rhythms themselves can affect mood. Light exposure or sleep manipulation which affects the circadian clock are now known to affect mood as well (Blume et al., 2019). For instance, chronotherapy using sleep deprivation, sleep phase advances, and bright light therapy has rapid antidepressant action in suicidal patients (Sahlem et al., 2014). Some medications, such as fluoxetine and lithium, used to treat MDD and bipolar disorder also affect the clock (Sprouse et al., 2006; McCarthy et al., 2013). Finally, the circadian clock regulates mood even in healthy subjects, as revealed by pronounced circadian rhythms of mood and reward motivation under controlled conditions (McClung, 2007; Logan and McClung, 2018). This is not a surprise considering that nearly half of all mammalian genes are expressed rhythmically in one or more tissues (Takahashi et al., 2008). Indeed, neuronal physiology and behavior are organized as a daily program that allows appropriate timing of functions and coordinated anticipation of the environmental 24 h day/night cycle (Andreatta and Allen, 2021).

In individual cells, the circadian clock regulates the expression of many genes critical for cell physiology and metabolism (Takahashi et al., 2008). At the molecular level, circadian rhythms are regulated by transcriptional–translational feedback loop where core transcriptional activators brain and muscle ARNT-like protein 1 (Bmal1), the circadian locomotor output cycles kaput (Clock), and the closely related neuronal PAS domain protein 2 (Npas2) bind at the E-box elements to modulate the expression of several genes, including period (Per) and cryptochrome (Cry), which, once translated, inhibit their own transcription (Astiz et al., 2019). Additional components of the feedback loop, such as Nr1d1/2 and Rora genes regulating Rev-erbs and Rors expression contribute to the precision and robustness of the clock (Preitner et al., 2002). These genes have been most studied in the suprachiasmatic nucleus (SCN), a master pacemaker region (Astiz et al., 2019). Several studies have shown association of clock gene polymorphisms with mood disorders, including associations of: MDD with Per2, Cry1, and Rora; seasonal affective disorder with Bmal1, Per2, Cry2, and Npas2; and bipolar disorder with Clock, Bmal1, Per3, Rev-erbα, and Rorb (McCarthy and Welsh, 2012). It is important to note that the expression of these genes varies within the brain. For example, Clock seems to be ubiquitously expressed in the brain while Npas2 is enhanced in the forebrain, particularly the nucleus accumbens (NAc) (Garcia et al., 2000), an important region regulating mood and reward (Lobo and Nestler, 2011; Russo and Nestler, 2013). Further support for a role of molecular clocks in mood disorders comes from human postmortem brain analyses which show significant alterations in the diurnal variation of the circadian clock genes in extra-SCN brain regions, including the NAc, amygdala, prefrontal cortex (PFC) and hippocampus in patients diagnosed with MDD (Li J. Z. et al., 2013).

At the cellular and synaptic level, clock genes or alterations in lighting conditions can alter excitability or synaptic activity in many regions that are also involved in anxiety, mood disorders, and reward (Logan and McClung, 2018; Andreatta and Allen, 2021). Despite the well-known circuit, cellular, and synaptic changes linked to depression-related behavior (Pittenger and Duman, 2008; Russo and Nestler, 2013; Francis and Lobo, 2017; Fox and Lobo, 2019), much less is known about how circadian clocks interact with these systems to either improve or in some cases induce mood-related disorders and anxiety. Current published work on reward-related behaviors may also provide information on how clock genes might drive alterations in affective states (Parekh and McClung, 2016). Clock genes can drive cell-specific changes in the NAc and dramatically alter responses to stress or other stimuli (Parekh et al., 2019; Porcu et al., 2020). Circadian clock alterations can contribute to enhancement or attenuation of excitatory (glutamatergic) or inhibitory (GABAergic) drive or their balance (Chellappa et al., 2016) through changes in synaptic plasticity or intrinsic properties that govern firing patterns of specific neuronal subtypes. In addition, lighting conditions, time of day, or manipulation of clock genes can affect synthesis or release of neuromodulators (e.g., ventral tegmental area (VTA) dopamine and serotonin) (Malek et al., 2007; Ferris et al., 2014). Altogether, these changes can have profound effects on how the brain processes information and promotes reaction to stimuli that underlie the genesis of mood and anxiety alterations.

These tissue-specific or cell-type specific patterns of expression and activity may account for differential roles of clock genes in mood and anxiety. With the advance of new tools, scientists have started to investigate circadian modulation (Table 1) and clock gene manipulation (Table 2) in different brain circuits and neuronal cell types in preclinical studies, which is a necessary step to advance our understanding on the mechanism underlying these disorders. Here, we discuss preclinical and clinical work showing circadian clock regulation in different neuronal populations and their role in mood-related phenotypes and speculate on how alterations in circadian clock may contribute to mood and anxiety-related disorders. We focus on the brain regions which have undergone more detailed assessment of circadian changes and their relationship to mood or anxiety and provide current information on known cell types that regulate these outcomes.

**Table 1:** Effects of circadian environmental manipulations on molecular and behavioral phenotypes.

Manipulation	Affected region/system	Cell-type	Species	Molecular/cellular phenotype	Behavioral phenotype	Reference
Lesions	SCN	N/A	Rat	N/A	Reduced immobility in FST; Decreased behavioral despair	Arushanyan and Popov (1995) and Tataroǧlu et al. (2004)
T22 light/dark cycle	SCN	N/A	Rat	Forced desynchrony of neuronal oscillators	Increased immobility in FST	Ben-Hamo et al. (2017)
Dim light at night	SCN	N/A	Mouse	Altered clock genes expression	Altered feeding behavior and increased body weight	Fonken et al. (2013)
Constant light	N/A	N/A	Mouse	Glucocorticoids concentration	Increasing depressive-like and decreasing anxiety-like responses	Fonken et al. (2009)
3 h light in the subjective night phase	SCN	GABA and VIP	Mouse	Changes in mPSCs frequencies	N/A	Cheng et al. (2018)
Long/short photoperiod	SCN	GABA, VIP, NMS	Mouse	Phase distribution, neurotransmitter switching	Circadian locomotor activity	VanderLeest et al. (2007), Evans et al. (2013), and Porcu et al. (2022)
Long/short photoperiod	PVN	SST/ DA, CRH	Mouse	Neurotransmitter switching, CRF release, neuronal activity	Depressive-like and decreasing anxiety-like responses	Dulcis et al. (2013) and Porcu et al. (2022)
Chronic mild stress	SCN	N/A	Mouse	Altered clock gene expression	Increased anxiety, depression-related behaviors	Logan et al. (2015)
Optogenetic activation	SCN	GABA	Mouse	Active phase resulted in a shortened period	Enhanced anxiety responses.	Vadnie et al. (2022)
Electrical stimulation of DNR	SCN	N/A	Hamster	Reduces light-induced fos protein	Causes circadian activity rhythm phase shifts	Morin and Meyer-Bernstein (1999)
SSRI	SCN	N/A	Rat	Phase advance in neuronal firing	N/A	Sprouse et al. (2006)
Chronic mild stress	NAc	N/A	Mouse	Altered clock gene expression	Increased anxiety and depression-like behavior	Logan et al. (2015)
Learned helplessness	NAc	N/A	Mouse	Less circadian rhythmicity and de-phased single-cell rhythms	Depression-like behavior	Landgraf et al. (2016a,b)
Chronic social defeat stress	NAc	N/A	Mouse	Alteration Per1 and Per2 expression	Increased anxiety responses	Spencer et al. (2013)
Chronic stress	NAc	N/A	Mouse	Increased Npas2 mRNA	Changes in forced swim test	Ozburn et al. (2017)
Clomipramine (CLI) injection	LHb	N/A	Mouse	Decreased Per2 expression	Depression-like behaviors	Li et al. (2021)
30 min Light pulse at ZT22	LHb	N/A	Mouse	Activation of Per1 expression	Alters despair behaviors	Olejniczak et al. (2021)
T7 cycle	PHb	N/A	Mouse	Altered Per2 and c-Fos expression	Increased depression-like behaviors	Fernandez et al. (2018)
3 h light at night for 3 weeks	PHb	N/A	Mouse	Altered circadian rhythm	Increased depression-like behaviors	An et al. (2020)
Retinal input ablation	DNR	N/A	Gerbils	Altered serotonin release	Potentiates depressive-like behavior	Ren et al. (2013)
Dim light at night	Hippocampus	N/A	Rat	Morphological changes including dendritic length, spine changes, and arbor reductions	Induced depression-like behaviors	Fonken et al. (2012)
Chronic mild stress	Hippocampus	N/A	Rat	Increases CDK5 activity	Induced depressive-like behavior	Yeung and Treit (2012)
Chemogenetic activation of ipRGCs	BLA, CEA,NAc,SCN	N/A	Mouse	Evoked circadian phase resetting and pupil constriction and induced neuronal activation	Increased anxiety-like behaviors in the elevated plus maze and open field test	Milosavljevic et al. (2016)
Chronic mild stress	BLA	N/A	Mouse	Disturbed diurnal oscillation of Clock, Cry2, Per1, Per3, Id2, Rev-erbα, Ror-β and Ror-γ expression	Anhedonic behavior	Savalli et al. (2015a,b)
Short photoperiod	BLA	N/A	White-footed mouse	Increased dendritic spine density	Increased fear memory in an auditory-cued fear conditioning test	Walton et al. (2012)

**Table 2:** Effect of clock gene manipulation on molecular and behavioral phenotypes.

Manipulation	Affected region/system	Cell-type	Species	Molecular/cellular phenotype	Behavioral phenotype	Reference
Chrono-KO	SCN	AVP	Mouse	Larger corticosterone response, abnormal glucocorticoid levels	N/A	Goriki et al. (2014)
Clock-Δ19	SCN	N/A	Mouse	Altered circadian rhythm	Increased motivation for rewarding stimuli, decreased depression-like behavior in the forced swim test and learned helplessness tests	Easton et al. (2003) and Roybal et al. (2007)
Per1-Per2 knock-out	N/A	N/A	Mouse	N/A	Depression-like behavior in the forced swim test	Spencer et al. (2013)
Cry1 or Cry2 single-KO and Cry1/2 double-KO	N/A	N/A	Mouse	N/A	Increased anxiety states but not depression-like behaviors	De Bundel et al. (2013)
Cry1/2 double-KO	N/A	N/A	Mouse	N/A	Increased anhedonia	Savalli et al. (2015a,b)
Cry2-KO	N/A	N/A	Mouse	N/A	Reduced despair-like behavior and increased anhedonia	Sokolowska et al. (2021)
Bmal1-KD	SCN	N/A	Mouse	Reduced rhythmicity	Increased behavioral despair, anxiety-like behavior, and helplessness compared to control	Landgraf et al. (2016a,b)
SOX2-KO	SCN	GABA	Mouse	Altered clock gene and neuropeptide expression	Less explorative behavior in the elevated plus maze, increased immobility time in the forced swimming test and tail suspension test, and decreased sucrose preference compared to control mice	Cheng et al. (2019)
CLOCK short-hairpin RNA	VTA	N/A	Mouse	DA release into the NAc and downregulation of GABAergic genes	N/A	Mukherjee et al. (2010)
Cck-KD	VTA	N/A	Mouse	N/A	Manic-like behavioral phenotype	Arey et al. (2014)
Clock-Δ19	VTA	N/A	Mouse	Increased TH mRNA, reduced cholecystokinin mRNA, increased dopamine neuron function as evidenced by increased glutamatergic tone as well as increased mean firing rate and bursting of DA neurons	Increased anxiety-related behavior	McClung et al. (2005) and Coque et al. (2011)
REV-ERBα deletion	VTA	N/A	Mouse	Repressed TH gene transcription by competing with nuclear receptor-related 1 protein	Hyperdopaminergic state and mania-like behavior	Chung et al. (2014)
Clock-Δ19	NAc	N/A	Mouse	Deficits in low-gamma and single-neuron phase coupling, dendritic morphology and reduced GluR1 expression	Increase exploratory drive	Dzirasa et al. (2010)
Clock-Δ19	NAc	N/A	Mouse	Reduced functional synaptic response in MSN, decrease protein levels and the rhythm of GRIA1 in MSN	Exploratory drive and reward sensitivity	Parekh et al. (2017)
NR1D1-KD	NAc	N/A	Mouse	Changes in circadian rhythm, including upregulation of Per1 and Per2 genes, and histone deacetylase genes	Enhanced social interaction and reduced anxiety	Zhao and Gammie (2018)
CR1/CRY2-KD	NAc	D1R-MSN	Mouse	Increased MSN neuronal activation at night.	Reduced susceptibility to helplessness	Porcu et al. (2020)
Per1/Per2-KD	NAc	N/A	Mouse	N/A	Regulation of anxiety related behavior	Spencer et al. (2013)
Npas2-KD	NAc	N/A	Mouse	Alters expression of GABA-A receptor subunits, prevents diazepam-induced potentiation of IPSC amplitude in MSNs	Reduced anxiety-like behavior	Ozburn et al. (2017)
Npas2-KD	NAc	D1R-MSN	Mouse	Blocked cocaine-induced enhancement of synaptic strength and glutamatergic transmission specifically onto D1R-MSN	Reduced cocaine conditioned place preference	Parekh et al. (2019)
Per2 deletion	NAc	Glia cells	Mouse	Increased GABA transporter 2 and dopamine receptor D3 mRNA and reduced glutamate levels	Reduced despair and anxiety	Martini et al. (2021)
Bmal1 deletion	NAc	Astrocytes	Mouse	Complex changes in AMPA receptor function and increased expression of AMPA and NMDA receptor subunits	Enhanced reward related behaviors including motivation for food and exploratory behavior	Becker-Krail et al. (2022a,b)
Per2-KD	LHb	N/A	Mouse	N/A	Induced depression-related behavior	Li et al. (2021)
Per1-KO	LHb	N/A	Mouse	N/A	Suppressed the beneficial effects of light on mood	Olejniczak et al. (2021)
SST -KO	BLA	SST	Mouse	N/A	Lack of circadian fluctuation of anxiety-like behavior	Albrecht et al. (2013)

## 2. Suprachiasmatic nucleus

The SCN is well known to be the circadian pacemaker of the brain and only recent evidence implicates it in mood regulation (Vadnie and McClung, 2017). The SCN is a bilateral nucleus in the hypothalamus composed by ~20,000 neurons which express the neurotransmitter gamma-aminobutyric acid (GABA) and multiple neuropeptides including: neuromedin S (NMS), arginine vasopressin (AVP), vasoactive intestinal polypeptide (VIP), and gastrin-releasing peptide (GRP) (Rosenwasser and Turek, 2010). NMS is expressed in ~40% of all SCN neurons (Lee et al., 2015), AVP-expressing neurons are localized in the dorsal shell region, and VIP and GRP neurons are found in the ventral core of the SCN (Romijn et al., 1997; Moore et al., 2002). VIP, GABA, and NMS maintain synchronization among SCN neurons, produce a coherent circadian rhythm and transmit output signals to other brain regions (Mori et al., 2005; Lee et al., 2015).

Mechanisms underlying circadian clock disruption (e.g., light phase variations, genetic knockout) in the SCN and its effect on mood regulation have been evaluated in rodent models. Initial studies assessed mood-related behaviors in a non-cell type specific manner by broadly lesioning the SCN or circadian forced desynchrony through light/dark cycle manipulation. These initial studies showed largely different results with SCN lesions causing less immobility in the forced swim test (Arushanyan and Popov, 1995; Tataroǧlu et al., 2004) and with artificially short light dark/cycle (22 h instead of 24 h cycle) inducing depressive phenotypes during the animal’s dark (active) phase (Hamo et al., 2016). However, with this methodology, it’s unclear whether circadian or non-circadian changes led to abnormal mood-related behaviors. Recent work has provided more insight into the role of SCN using altering light phase, behavioral stress, genetic manipulation (i.e., knockdown, knockout, or overexpression) of peptides or clock genes, or selective cell-type activation.

Since light is a strong environmental cue for the mammalian circadian clock that synchronizes an organism’s biological rhythms (also known as a zeitgeber) (Golombek and Rosenstein, 2010), and the SCN is receptive to inputs directly responding to light in the retina, previous studies have assessed the effects of altered light environment on SCN activity and mood-related behaviors (Legates et al., 2014). Dim light at night perturbed clock gene expression rhythms in the SCN (Fonken et al., 2013), and mice exposed to constant light showed increased depressive-like behavior, decreased anxiety-like responses and altered glucocorticoid concentration (Fonken et al., 2009). Since daily corticosterone release depends on coordinated clock genes expression and neuronal activity rhythms in both VIP neurons in the SCN and CRH neurons in the paraventricular nucleus of the hypothalamus (PVN) (Jones et al., 2021), altered mood responses might be mediated by the SCN via glucocorticoids release. Importantly, circulating corticosteroids have a wide range of physiological effects and have been implicated in stress response and depression (Arborelius et al., 1999).

Excitatory and inhibitory activity in the SCN undergoes daily regulation, with increased glutamate miniature excitatory postsynaptic current (mEPSC) frequency and GABAergic miniature inhibitory postsynaptic current (mIPSC frequency) in VIP neurons in the dark phase (Cheng et al., 2018). Increasing excitatory and inhibitory balance on these cells can scale up overall activity of these VIP cells at night thereby increasing VIP release. VIP from these neurons is critical for maintaining and synchronizing rhythms in the SCN (Evans et al., 2013). Since altered rhythms can lead to susceptibility to mood and anxiety disorders, it is likely perturbations to VIP release will affect the expression of mood changes or responses to events that drive mood. Three hours of light exposure at the beginning of the subjective night suppressed the glutamateric and GABAergic changes in mEPSC and mIPSC frequencies, while exposure to a skeleton photoperiod did not affect mEPSC or mIPSC frequencies between the subjective day and night (Cheng et al., 2018). This study may implicate afferent glutamatergic and local GABAergic synaptic transmission on VIP neurons within the SCN in mood and anxiety outcomes, particularly in cases of perturbed start to the dark phase. Currently, little is known about how this synaptic activity is altered in mood disorders and anxiety.

Additional behavioral studies using unique light protocols provide some insight into SCN function in mood and anxiety. Short photoperiod exposure was found to induce depressive-like and anxiety-like responses in nocturnal hamsters (Pyter and Nelson, 2006), representing an animal model of seasonal affective disorder. Interestingly, short and long photoperiod exposure alters the phase distribution of SCN neuronal activity (VanderLeest et al., 2007; Evans et al., 2013) as well as VIP and NMS expression in the SCN (Porcu et al., 2022). Rats exposed to a long photoperiod showed increased anxiety and depression-like behaviors with a parallel decrease in dopaminergic neurons in the PVN (Dulcis et al., 2013) that receive input from the SCN (Romanov et al., 2017; Porcu et al., 2022), suggesting an important role of the SCN in mediating seasonal changes in light-dependent effects on mood. Altering the light phase provides a direct way of assessing how circadian clock alter behavior and may be a stressor on its own, particularly in nocturnal rodents. Chronic stress paradigms, however, are a useful way to induce depression-like and anxiety-like behavior in animals and examine resultant effects on a variety of systems. Very few studies have been done to examine the effects of depression models on SCN activity or clock gene expression (Logan et al., 2015; Landgraf et al., 2016b). Logan and colleagues showed that chronic unpredictable stress affected clock gene expression in the SCN of mice showing increased anxiety, depression-related behaviors, and physiological responses akin to human depressed patients (Logan et al., 2015). Circadian clock disturbances were also observed in other brain regions, such as the nucleus accumbens (Logan et al., 2015; Landgraf et al., 2016b). Because the SCN sends multi-synaptic connections to the VTA which projects to the NAc, the master circadian pacemaker might regulate mood and anxiety through modulation of mesolimbic circuits (see *Ventral tegmental area and Nucleus Accumbens* section).

Knockout or genetic manipulation of clock genes have provided the most specific information about how specific cell-types affect mood and anxiety. Several studies have investigated the effects of VIP, AVP, and NMS peptide knockdown and/or knockout on circadian behaviors and SCN circuit synchronization (Ono et al., 2021). However, changes in mood-related behaviors were not reported. Studies in humans have shown a decrease in SCN AVP neurons in patients with depression (Zhou et al., 2001), which may diminish neuronal coupling and suppress output signals that participate in synchronizing daily rhythms. *In vivo* studies of SCN AVP neuron-specific *Chrono* KO in mice reported longer circadian periods in their activity rhythms and increased corticosterone response after acute stress (Goriki et al., 2014). Since AVP neurons regulates hypothalamo–pituitary–adrenal (HPA) axis function during stress (Kovacs et al. 200) and abnormal corticoid levels are associated with mood disorders (Landgraf et al., 2015), CHRONO could have a crucial role in regulating SCN AVP activity which in turn affect glucocorticoid release and mood.

Rhythms in neuronal activity and neurotransmitter expression in the SCN are regulated by the clock genes. Therefore, altered circadian clock gene expression could lead to neuron physiology disruptions, which ultimately affect peripheral biological systems under the SCN’s influence. Indeed, Clock-Δ19 mice, which contain a somatic dominant-negative mutation of the gene Clock, showed increased motivation for rewarding stimuli, decreased depression-like behavior in the forced swim test, and learned helplessness tests (Easton et al., 2003; Roybal et al., 2007). In addition, Clock-Δ19 mice are similar to human mania, showing hyperactivity and decreased sleep. Treatment with lithium, a mood stabilizer widely used in treating bipolar disorder suppressed the exploratory phenotype in these mice (Roybal et al., 2007). Mice lacking both Per1 and Per2 (Bae et al., 2001) showed robust increase in anxiety and depression phenotypes (Spencer et al., 2013). However, there are different observations on the effects of CRY1 and CRY2 on mood and anxiety. Indeed, while one study showed that Cry1 or Cry2 single-KO and Cry1/2 double-KO mice showed increased anxiety states, but not depression-like behaviors (De Bundel et al., 2013), another study reported Cry1/2 double-KO displayed increased anhedonia compared to WT mice (Savalli et al., 2015a). A recent study found no differences in anxiety or depression-related behaviors in Cry1/2 double-KO (Hühne et al., 2020). Interestingly, Cry2-KO mice showed reduced despair-like behavior and increased anhedonia, but not anxiety-like behaviors (Sokolowska et al., 2021).

It is important to note that all these mouse models used overexpression or knock out variants of clock genes which are neither cell- or region-specific, therefore the observed effects on mood and anxiety might be due to indirect effects of the mutation on several unrelated processes in other brain areas. The best evidence for a SCN-specific effect of clock genes in mood regulation, comes from Landgraf and colleagues which induced an attenuation of SCN circadian rhythms by short hairpin RNA-induced knockdown (KD) of Bmal1. SCN-Bmal1-KD mice showed increased behavioral despair, anxiety-like behavior, and helplessness compared to control, while no changes were observed in hedonic behavior and spatial preference between SCN-Bmal1-KD mice and control mice (Landgraf et al., 2016a,b).

Molecular players regulating circadian gene expression or activity can also modulate neuronal physiology within the SCN circuit affecting behavior. The transcription factor SRY (sex-determining region Y)-box 2 (SOX2) positively regulates transcription of the core clock gene Per2 (Cheng et al., 2019). Using GABAergic-restricted conditional knockout (cKO) of SOX2 in the SCN, Cheng and colleagues found severely disrupts rhythms of locomotor activity in mice, reduces *Per2* and decreases neuropeptides and expression of neuropeptide receptors within the SCN (Cheng et al., 2019). Interestingly, the authors later showed that the Sox2-cKO mice exhibited less explorative behavior in the elevated plus maze, increased immobility time in the forced swim test and tail suspension test, and decreased sucrose preference compared to control mice (Boehler et al., 2021), suggestive of increased anxiety and depression related behavior. Altogether these studies demonstrate that abolishing SOX2 expression in GABAergic neurons of the SCN perturbs behaviors that are associated with anxiety, depression, and motivation (or anhedonia) in mice likely by dysregulation of cellular timekeeping within SCN neurons and neuropeptide release regulating temporal information to other brain regions.

A recent study also highlights the important role of SCN GABAergic neuronal activation in the regulation of anxiety states in mice. To investigate the role of the SCN in regulating depressive- and anxiety-like behavior in mice, Vadnie and colleagues used optogenetic stimulation paradigms to chronically manipulate the GABAergic neural activity in the SCN. Chronic stimulation of the SCN GABA neurons late in the active phase resulted in a shortened period and dampened amplitude of locomotor activity rhythms and increased anxiety-like behavior in the elevated plus maze. Interestingly, acute SCN GABA neuron activation also enhanced anxiety responses. The authors conclude that dampening of rhythms in SCN GABA neurons can increase anxiety-like behavior in mice (Vadnie et al., 2022).

Altogether these studies are an important step into our understanding of the role of the SCN in mood-related behaviors, however the contribution of different SCN cell-types on depression and anxiety-like phenotypes is still unknown. Considering the heterogeneity of neurons in the SCN and their neural projections, future studies using cell-type specific approaches for clock genes and activity manipulation are needed to unveil the contribution of SCN neuronal subpopulation in mood regulation.

## 3. Ventral tegmental area and nucleus accumbens

Brain regions such as VTA and the NAc, central components of the highly interconnected mesolimbic dopamine circuit and critical regions for controlling reward and mood, exhibit their own specific rhythmic organization controlled by local clocks and clock-controlled inputs (DePoy et al., 2017). In the past few years, several studies in both rodents and humans have shown remarkable clock gene rhythms in both the VTA and NAc (Becker-Krail et al., 2022b). Early studies have reported circadian transcriptional regulation of tyrosine hydroxylase and monoamine oxidase in the VTA, which are enzymes crucial for DA synthesis and deactivation, respectively (Hampp et al., 2008; Logan et al., 2018). Consequently, an intra-diurnal rhythmic pattern of VTA DA neuronal activity was observed (Domínguez-López et al., 2014). Both phasic and tonic DA release is modulated by the circadian clock through the DA transporter and tyrosine hydroxylase (TH) expression, which regulates the diurnal variation of DA in the NAc (Ferris et al., 2014). Additionally, chronic stress, a risk factor for depression, alters the amplitude or phase of clock gene expression in the NAc (Logan et al., 2015). Specific cell types within each region regulate these rhythms and/or are receptive to rhythms from other regions.

Studies examining VTA clock-related changes related to mood have primarily focused on dopamine neurons or dopamine release. Clock-Δ19 mice showed altered gene expression of several key dopamine-related within the VTA including increased TH mRNA and reduced cholecystokinin (Cck) mRNA expression. These changes corresponded to increased dopamine neuron function as evidenced by increased glutamatergic tone as well as increased mean firing rate and bursting of DA neurons, which could be rescued by chronic lithium treatment (McClung et al., 2005; Coque et al., 2011). In agreement with these studies, Mukherjee and colleagues found that local infusion of CLOCK short-hairpin RNA in the VTA recapitulates the increase of DA release into the NAc (Coque et al., 2011), suggesting Clock-Δ19 changes were due to local VTA disruption of the CLOCK gene. Furthermore, VTA knockdown of Cck, a direct transcriptional target of CLOCK, is sufficient to recapitulate a manic-like behavioral phenotype (Arey et al., 2014). Increased sensitivity to ethanol and ketamine in Clock-Δ19 mice was also observed in VTA-specific Clock-knockdown mice (Ozburn et al., 2013). Clock-Δ19 mice and Clock-knockdown mice also showed a downregulation of GABAergic genes in the VTA (Mukherjee et al., 2010), suggesting a potential reduction of inhibitory control and disinhibition of DA neuronal activity. Altogether these studies support the unique function of CLOCK in the VTA in contrast to its widespread circadian transcriptional role in the SCN and other brain regions.

Clinical evidence implicates the involvement of the circadian nuclear receptor REV-ERBα in mood regulation. REV-ERBα is encoded by the NR1D1 gene, and genetic variations in human NR1D1 loci are associated with bipolar disorder onset and responsiveness to lithium (Kripke et al., 2009; Severino et al., 2009; Etain et al., 2011). Chung and colleagues found that genetic deletion of the REV-ERBα gene or pharmacological inhibition of REV-ERBα activity in the VTA induced hyperdopaminergic state and mania-like behavior (Chung et al., 2014). They also showed that REV-ERBα repressed TH gene transcription by competing with nuclear receptor-related 1 protein (NURR1), which altered circadian TH expression via a target-dependent antagonistic mechanism.

VTA neurons project to the NAc which plays a crucial role in regulating motivation and reward functions (Nestler, 2015). Dopamine signals in both the dorsal and ventral striatum are critical for reward and motivation. Recently, Stowe and colleagues showed diurnal rhythms in rapid dopamine signaling in the striatum which are mediated by time-of-day shifts in cholinergic interneuron control of dopamine. These systems work together to mediate time-of-day changes in conditioned responses to reward-associated cues (Stowe et al., 2022). The major NAc projection neurons that regulate these behaviors are GABA and peptide expressing neurons, also called medium spiny neurons (MSNs). The MSNs are classified by their DA receptor expression dopamine type 1 receptor or dopamine type 2 receptor, D1R-MSN or D2R-MSN, respectively. Activity of these two neuronal populations promotes dichotomous behavioral outcomes caused via cellular plasticity, subcellular signaling pathways, and genetic expression, playing a key role in regulating mood (Francis et al., 2015; Francis and Lobo, 2017). In most cases, enhancing D1R-MSN activity generates positive mood and reward-related outcomes while D2R-MSNs are responsible for blunting these outcomes (Lobo et al., 2010; Francis and Lobo, 2017). Suppression of glutamatergic activity to D1R-MSNs promotes anhedonic phenotypes (Lim et al., 2012; Francis et al., 2015; Francis and Lobo, 2017; LeGates et al., 2018; Francis et al., 2019). Rhythmic extracellular changes in DA, glutamate, and GABA are seen in the striatum and NAc (Castañeda et al., 2004), all neurotransmitters which play major roles in mood and stress-related outcomes. Studies in humans and animal models have shown clock disruptions in the NAc associated with mood dysregulation. Li and colleagues found remarkably weaker daily rhythms of clock gene expression in post-mortem human brains with a diagnosis of major depression disorder compared to healthy subjects (Li J. Z. et al., 2013). In rodents, chronic mild stress affects clock gene expression in the NAc associated with increased anxiety and depression-like behaviors (Logan et al., 2015). NAc brain slices from helpless mice showed less circadian rhythmicity and dephased single-cell rhythms compared to those from resilient mice (Landgraf et al., 2016b).

Electrophysiological recording in Clock-Δ19 mice showed profound deficits in low-gamma and single-neuron phase coupling in the NAc. Chronic lithium treatment ameliorated these neuronal phase-locking deficits. In addition, the authors found changes in dendritic morphology and reduced GluR1 expression in the NAc of Clock-Δ19 mice compared to WT littermates (Dzirasa et al., 2010). This data suggest that disruption of the Clock gene is sufficient to induce biochemical, morphological, and neurophysiological changes in NAc microcircuit affecting mood. Using whole-cell patch-clamp electrophysiology, Parekh and colleagues revealed that the Clock-Δ19 mice had reduced functional synaptic response in NAc neurons (Parekh et al., 2019), which can be caused by a loss of glutamatergic receptor function. Indeed, NAc surface protein levels and the rhythm of AMPA receptor subunit GRIA1 are decreased in the Clock-Δ19 mice and overexpression of functional Gria1 in the NAc normalizes increased exploratory drive and reward sensitivity behavior (Parekh et al., 2017). GRIA1 expression changes and surface receptor expression are suggestive of AMPA receptor plasticity. Since D1R-MSNs are required for reward related behavior (Lobo et al., 2010) and reduced AMPA receptor function in D1R-MSNs underlies reward and anhedonia deficits (Lim et al., 2012; LeGates et al., 2018), it is possible that D1R-MSNs could drive these changes observed in Clock-Δ19 mice. In addition, altered expression of NMDA receptor subunits can promote circadian changes leading to mood or anxiety alterations. For instance, NR1D1 knockdown females’ mice showed changes in the circadian clock, including upregulation of Per1 and Per2 genes, and histone deacetylase genes (Zhao and Gammie, 2018). Knockdown of NR1D1 gene in the NAc enhanced social interaction and reduced anxiety in female mice but not in male mice (Zhao and Gammie, 2018).

Recently, we found that mice exhibiting helplessness showed altered circadian clock function and high nighttime expression of CRY in the NAc, associated with decreased activation of D1R-MSNs (Porcu et al., 2020). Consistent with these observations, D1R-MSN–specific Cry-knockdown in the NAc reduced susceptibility to helplessness and increased NAc neuronal activation at night. Altered circadian clock and Cry mRNA expression were also observed in human fibroblasts from major depressive disorder patients (Porcu et al., 2020). Chronic social defeat stress induced mPer1 and mPer2 expression in the NAc and elective Per1 and Per2 knockdown in the NAc increased anxiety-like behavior as seen in the mPer1/mPer2 mutant animals (Spencer et al., 2013). Chronic treatment with the antidepressant fluoxetine, which is known to selectively enhance excitatory synaptic function on D1R-MSNs in stressed mice (LeGates et al., 2018), normalized *Per* gene expression towards wild-type levels in the NAc (Spencer et al., 2013). NPAS2, a paralog of CLOCK, which heterodimerize with BMAL1 to activate repressor genes such as Per and Cry is highly expressed in the NAc, specifically in D1R-MSN. Ozburn and colleagues found that Npas2-KO mice displayed anxiety-like behavior in elevated plus maze, light/dark box, and open field tests compared to WT mice (Ozburn et al., 2017). NAc-specific knockdown of Npas2 blocked cocaine-induced enhancement of synaptic strength and glutamatergic transmission specifically onto D1R-MSNs. D1R-MSN–specific Npas2-knockdown in the NAc reduced cocaine conditioned place preference (Parekh et al., 2019) We argue that, due to large variations in phasic dopamine through D1Rs and smaller changes in tonic dopamine, defined alterations in MSN intrinsic or synaptic activity, and changes in receptor expression, D1R-MSNs may be the primary cell targets that contribute to circadian-driven mood-related outcomes.

Altering clock genes in NAc glial cells can have profound effects on activity and mood-related behavior. Astrocytes are a highly abundant glial cell type essential for regulation of glutamate levels via glutamate uptake and release and neurometabolic homeostasis (Scofield and Kalivas, 2014; Kim et al., 2018). Importantly, astrocytes also contain a circadian molecular clock and clock genes regulate astrocyte functions (Prolo et al., 2005; Brancaccio et al., 2017). Deletion of Per2 in mouse astrocytes reduced despair and anxiety associated with increased GABA transporter 2 and dopamine receptor D3 mRNA and reduced glutamate levels in the NAc (Martini et al., 2021). However, neuronal Per2 knockout also reduces despair, but not anxiety responses. Astrocytic Bmal1 deletion in the NAc did not affect despair or anxiety (Martini et al., 2021). A recent study demonstrated that astrocytes in the NAc showed strong molecular rhythms which regulates NAc metabolic homeostasis and behavior. Genetic disruption of NAc astrocyte molecular clock function via NAc-specific Bmal1-KO enhanced reward related behaviors including motivation for food and exploratory behavior (Becker-Krail et al., 2022a,b). These behaviors were putatively facilitated by complex changes in AMPA receptor function and increased expression of AMPA and NMDA receptor subunits. Altogether, these results suggest an important role of circadian clock in different cell subtypes in the NAc in the reward-related behavior and mood regulation.

## 4. Habenula and thalamus

The habenula is adjacent to and communicates with thalamic regions which receive direct projections from the retina. The lateral habenula (LHb) has its own circadian rhythmicity as evidenced by *ex vivo* slice preparations. The LHb neurons exhibit intrinsic daily changes in their firing rate, with higher activity levels during the middle to late day and early night (Zhao and Rusak, 2005; Sakhi et al., 2014a). Consistent with this, the daily rhythms of neuronal LHb activity is absent in LHb brain slices from mice lacking a functional molecular circadian clock (Cry1,Cry2-KO mice) (Sakhi et al., 2014b). The LHb is a brain region that plays a crucial role in processing reward and aversion signals, as well as in regulating various behavioral and emotional states (Stamatakis and Stuber, 2012) and activity of this region causes anhedonia and helplessness through CamKII neurons (Li J. Z. et al., 2013; Li K. et al., 2013; Yang et al., 2018). The finding of daily fluctuations in the firing rate of LHb neurons implies that the timing of neural activity in this region may have important implications for its downstream targets in the midbrain. These midbrain targets include regions such as the ventral tegmental area (VTA) and the dorsal raphe nucleus (DRN), which are involved in reward processing, motivation, and mood regulation (Quina et al., 2015). Further investigations are needed to fully understand the precise mechanisms underlying the daily changes in firing rate within LHb neurons and their impact on midbrain targets. Elucidating these mechanisms would provide valuable insights into the role of the LHb in temporal processing, reward modulation, and mood.

Of note, a previous study revealed that CaMKIIa LHb neurons received light information from thalamic ventral lateral geniculate nucleus and intergeniculate leaflet GABAergic neurons which are innervated by ipRGCS (Huang et al., 2019). Light at zeitgeber time (ZT) 22 showed to affect mood-related behaviors in mice by activating the clock gene Period1 (Per1) in the LHb. Indeed, selective silencing of the clock gene *Per2* in the LHb in rats induced depression-like behavior at night, (Li et al., 2021) and deletion of *Per1* in the LHb suppressed the beneficial effects of light on despair related behavior (Olejniczak et al., 2021) suggesting that *Per* genes may play an important role in the pathogenesis of depression. Modulation of LHb GABA neurons regulates depressive-like behaviors and provides a potential circuit mediating the effects of phototherapy in depression (Huang et al., 2019).

The perihabenular nucleus (pHb) of the dorsal thalamus is integrated into a distinctive circuitry with mood-regulating centers and has been shown to regulate the effects of light on affective behavior. Fernandez and colleagues were the first to reveal two distinct retinal-brain pathways that mediate the direct effects of light on mood and cognition. Irregular light environment increased activity of pHb neurons, sustained light-mediated induction of the immediate-early gene c-Fos, and an altered clock gene rhythmicity (Fernandez et al., 2018). Mood alterations associated with changes in the thalamic pHb, included increased immobility time in the tail suspension test and in the forced swim test, as well as reduced sucrose preference. These depression-like behaviors were no longer observed in Opn4Cre/+;Brn3bDTA mice, which lack ipRGCs projection to the pHb but not to the SCN (Fernandez et al., 2016). In agreement with this, a recent study confirmed an SCN-independent pathway linking ipRGCs to pHb in regulating light-induced mood responses (An et al., 2020). The authors found that light acutely activates pHb–NAc projections under circadian clock modulations. Using electrophysiological recordings, they showed that NAc-projecting dorsal pHb neurons were capable of sustained activity when injected with depolarizing currents at night, while the same amount of injected current failed to evoke sustained activity in these neurons during the light phase, suggesting that the circadian clock gates the excitability of NAc-projecting dorsal pHb neurons (An et al., 2020). Importantly, they showed that pHb–NAc projecting neurons mediate the depressive-like behaviors induced by light at night. This interconnectivity between circadian-related regions involved in mood highlights the complexity of mood regulating brain systems and provides a foundation for more cell-type specific or pathway manipulation to determine how these interconnected systems distinctly regulate behavior.

## 5. Dorsal raphe nucleus

The Dorsal Raphe Nucleus (DRN) has been implicated in circadian-driven mood disruption. The DRN supplies the brain with serotonin, including regions involved in circadian regulation such as the SCN. Loss of serotonin input through a SCN serotonin receptor knockout can alter SCN neuronal activity, affecting the period and amplitude of SCN rhythms (Ciarleglio et al., 2011). The DRN receives direct input from the retina (Hattar et al., 2006) and light primarily activates GABAergic cells within this region and a small percentage of serotonin neurons (Güler et al., 2008). In fact, suppression of retinal input to the DRN potentiates depressive-like behavior and altered serotonin release from this region (Ren et al., 2013). Like the VTA dopamine, DRN serotonin production is regulated through circadian rhythmic expression of the Tph2 gene which produces tryptophan hydroxylase, a enzyme necessary for the synthesis of serotonin (Malek et al., 2005). In addition, Tph2 expression is sensitive to glucocorticoids (Malek et al., 2007), which could affect daily rhythms of serotonin synthesis and release.

The DRN is thought to be critically involved in depression and/or anxiety related behavior due to the use of common drugs used to treat these ailments (selective serotonin reuptake inhibitors, SSRIs) that block serotonin reuptake. Several pharmacological studies have suggested that the large median raphe serotonergic projection to the circadian clock in the SCN may modulate the phase of circadian rhythms. Electrical stimulation of the median or dorsal raphe nuclei reduces light-induced fos protein in the SCN and causes phase shifts in circadian activity rhythms (Morin and Meyer-Bernstein, 1999). This phase shift may also be influenced by a disynaptic DRN to medial raphe nucleus circuit projecting to the SCN (Glass et al., 2003). Interestingly, SSRIs and other serotonin affecting drugs can influence circadian clocks by phase advance in SCN neuronal firing (Sprouse et al., 2006). In one study, immobility in the tail suspension test was more effectively reduced by administration of fluoxetine administration at the start of the light phase of rodents (inactive phase), while other antidepressants that target additional systems showed different profiles (Kawai et al., 2019). Advancement in understanding of how time of day administration of common antidepressants affect mood will provide a better means of developing chronopharmacology to provide more effective treatment schedules for these mood disorders.

## 6. Hippocampus and amygdala

The hippocampus is well known to show significant pathology in depression including alterations in neuronal structure, synaptic activity and plasticity, and neurogenesis (Duman and Monteggia, 2006) and volume and activity alterations in humans is a predictor of successful treatment (Bell-McGinty et al., 2002; Hallahan et al., 2011; Gerlach et al., 2022). In addition, many of these changes can be ameliorated with antidepressants (Thompson et al., 2015). Using *in vivo* recording approaches, more attention is being given to how neuronal synaptic plasticity, and particularly, how firing rates are modulated by the circadian clock. Primarily excitatory, glutamatergic neurons have been the primary focus of this region and several studies showed that the magnitude of hippocampal long-term potentiation (LTP) varies based on the time of day (Paul et al., 2020). In Schaffer collateral activation in CA1, LTP is larger when rodent slices were prepared at night as opposed to the day (Harris and Teyler, 1983; Chaudhury et al., 2005; Bowden et al., 2012) and time of day alters the ability to induce LTP or alters the size of LTP in the dentate gyrus (Bowden et al., 2012). This may suggest the active phase may facilitate more robust excitatory transmission to efferent regions like the NAc during the active phase of animals, and thus, would alter responses to stimuli that promote mood and anxiety. These changes are supported by reports that astrocytes retract their processes and uptake less glutamate during the dark phase (McCauley et al., 2020). Dentate gyrus (DG) neurons also exhibit a 24-h cycle of intrinsic excitability and synaptic recruitment mediated regulation of membrane currents, regulated by cell intrinsic molecular clock transcriptional machinery (Gonzalez et al., 2023). Additionally, dim light at night has the capability of producing depression-related behavior and morphological changes including dendritic length, spine changes, and arbor reductions in the DG (Fonken et al., 2012). Together, these alterations in synaptic and intrinsic activity in the hippocampus indicate that time of day can have profound impacts on how plasticity within this region can be more or less sensitive to environmental perturbations or stimuli that promote susceptibility to mood and anxiety disorders.

Hippocampal clock gene expression shows circadian rhythmicity (Jilg et al., 2010) and factors that regulate clock genes in the hippocampus can have significant effects on depression-related behavior. Cyclin-dependent-like kinase 5 (CDK5) is critically involved in regulating the circadian clock via CLOCK phosphorylation at the Thr-451 and Thr-461 residues which alters CLOCK stability and subcellular distribution, resulting in transcriptional activation of CLOCK (Kwak et al., 2013). Zhu and colleagues found that chronic mild stress in rats increases CDK5 activity in the hippocampus, particularly in the DG subregion. Increased CDK5 activity in the hippocampus induced depressive-like behavior while regional inhibition of CDK5 in DG but not in the CA1 or CA3 hippocampal subregions decreased the development of depressive-like behaviors in rats (Zhu et al., 2012). This suggests CDK5 expression in the DG may promote susceptibility to depressive-like behavior through regulation of circadian proteins such as CLOCK.

The primary focus of much of this work in the hippocampus has been on excitatory, glutamatergic neurons. However, some recent data sheds light on how inhibitory hippocampal neurons may be involved in circadian regulation of mood. Somatostatin (SST) neurons in the hippocampus co-express GABA and are primarily inhibitory (Honoré et al., 2021). Analyzing hippocampal tissue from a cohort of subjects with substance use disorder and subjects with major depression Valeri and colleagues found decreased expression of SST in subjects with substance use disorder and in subjects with major depression (Valeri et al., 2022). Clock gene Arntl, Nr1d1, Per2 and Cry2 expression in the hippocampus was increased in subjects with substance use disorder, while Arntl and Nr1d1 expression was decreased in subjects with major depression (Valeri et al., 2022). SST release and clock genes may represent key converging pathways involved in contextual memory processing in mood regulation, providing more evidence for circadian alterations in the hippocampus that are consequences of mood disorders.

Growing evidence indicates that expression of SST in the amygdala plays a key role in impulsive behavior and in modulation of anxiety (McDonald et al., 1995; Engin et al., 2008; Yeung and Treit, 2012; Albrecht et al., 2013). Early studies have suggested a link between SST and circadian rhythms in bipolar disorder, showing diurnal SST cerebrospinal fluid, which peaks in the morning (Rubinow, 1986). Consistent with this observation, postmortem human brains analysis revealed daily changes in SST neurons density and number in the amygdala in healthy subjects, which was altered in bipolar patients (Pantazopoulos et al., 2017). In mouse models, SST in the amygdala has been shown to mediate the modulatory influence of circadian clocks on innate anxiety (Albrecht and Stork, 2017). Per2 mRNA levels display pronounced circadian fluctuation over a 24 h period in the basolateral amygdala which is correlated with SST. Testing the mice during two time periods of their active phase, i.e., in the first or the second half of the dark phase of the light / dark cycle, Albrecht and colleagues found increased locomotor activity towards the second half of the dark phase that in an open field test and reduced anxiety-like behavior of SST+/+ mice during the second half of the dark phase compared to WT mice (Albrecht et al., 2013). Interestingly, chemogenetic activation of ipRGCs, which simulates the excitatory effects of bright light on this cell type in dark-housed mice, evoked circadian phase resetting and pupil constriction and induced neuronal activation in the SCN, NAc, and basolateral and central amygdala (Milosavljevic et al., 2016). These neurophysiological events were associated with increased anxiety-like behaviors in the elevated plus maze and open field test (Milosavljevic et al., 2016). In mice, chronic mild stress-induced anhedonic behavior was associated with disturbed diurnal oscillation of Clock, Cry2, Per1, Per3, Id2, Rev-erbα, Ror-β and Ror-γ expression of in the mouse basolateral amygdala (Savalli et al. 2015b). Altogether these data suggest that circadian effects on anxiety responses and mood regulation are linked to the natural activity rhythms in the limbic system, specifically in amygdala neurons. Indeed, Lamont and colleagues demonstrated that various subregions of the amygdala display daily oscillations in clock gene expression. Importantly, these oscillations are not solely regulated by the SCN, but are also influenced by hormonal and neurochemical changes that occur during different motivational and emotional states (Lamont et al., 2005). Overall, this research sheds light on the complex interplay between the molecular clock, hormonal influences, and emotional states within the amygdala. Further exploration of these mechanisms may provide valuable insights into the links between circadian rhythms and emotional regulation, potentially paving the way for novel therapeutic strategies targeting circadian clocks in the amygdala circuitry.

Blue wavelength light has been used successfully as treatment for certain mood disorders, recent evidence in humans revealed that acute blue light exposure (relative to green) increased responses to emotional stimuli in the temporal voice area of the temporal cortex and in the hippocampus. During emotional processing, the functional connectivity between the temporal voice area, the amygdala, and the hypothalamus was selectively enhanced after blue light exposure (Vandewalle et al., 2010). In addition, 30 min of blue light exposure in the morning for 3 weeks enhanced positive connectivity between the right amygdala and a region within the left dorsolateral PFC associated with greater decreases in negative mood for the blue compared to ambient light condition (Alkozei et al., 2021).

In white-footed mice, a small photoperiodic rodent, ten weeks of exposure to 8 h of light and 16 h of darkness (short-day photoperiod) increased fear memory in an auditory-cued fear conditioning test and increased dendritic spine density of the neurons of the basolateral amygdala, without affecting morphology of pyramidal neurons within the infralimbic region of the medial prefrontal cortex (Walton et al., 2012). Altogether these studies suggest that light and its spectral composition regulate emotional brain processing and identify a unique network merging affective and ambient light information. Future studies are needed to reveal the contribution of amygdala rhythmic functions and neuronal cell-type regulating light-induced emotional responses.

## 7. Conclusion and future directions

Circadian-driven gene expression and neuronal activity are heavily influenced by stress and stress itself can influence circadian dependent activity. Additionally, it is clear the convergence of these two systems can strongly influence susceptibility to mood and anxiety disorders. Despite this, extensive work is still necessary to define how circadian clocks, genes that underlie these rhythms, and their resultant effect on cell subtype selective cellular and circuit activity alter mood and anxiety.

It is important to mention that circadian clocks are also influenced by seasonal changes in the environment at cellular and molecular levels (i.e., daylength) (Garbazza and Benedetti, 2018). Failing to adjust to these seasonal changes may heighten the risk of mood and behavioral issues, as well as lead to poorer clinical outcomes in psychiatric disorders. Seasonal changes have been observed to alter brain function and psychiatric symptoms (Zhang and Volkow, 2023). Misalignment between various biological rhythms and seasonal changes in the environment might impact mood and stress responses through gene expression, neurotransmission, and hormone releases. Further research is needed to identify cell-type and circadian processes that contribute to the seasonality of psychiatric symptoms and vulnerability.

We have entered an era of technological expansion in areas that could affect rhythms and promote susceptibility to these disorders. For instance, modern technology like cell phones exposes humans to altered light cycles or wavelengths at inappropriate times of day (i.e., not in normal phase) and there is an increased prevalence of anxiety in depression (Tancredi et al., 2022). We and others propose these two factors are linked where altered light phases can lead to poor mood outcomes or anxiety. In addition, humans face increased psychosocial stress due to new technologies and social connectedness (Hensel et al., 2022). These stressors are likely to regulate our own internal rhythms and contribute to disease.

The work highlighted here provides an overview of the few known brain-region and cell-type selective players at the intersection of environmental stressors and circadian clocks. The field is young and requires significant work to tackle the challenges and provide understanding of how environmental or interoceptive factors interact to promote mood and anxiety disorders. We suggest a few future avenues for additional work. First, cell-type specific manipulation of clock genes and assessment of mood outcomes in stress-naive and stressed mice could provide a sufficient starting point to untangle the circadian players in mood. Next, we suggest examining how clock genes contribute to activity of these cell types through synaptic function or circuit firing dynamics. Lastly, we hope that this research can be extended further to develop chronotherapies or chronopharmacology for the treatment of mood disorders and anxiety.

## Author contributions

TF and AP contributed equally to the development, writing, and editing of the manuscript. All authors contributed to the article and approved the submitted version.

## Funding

This work was supported by the College of Pharmacy, Department of Drug Discovery and Biomedical Sciences at the University of South Carolina and NIH/NIMH R00MH123673 (TF) and NIH/NCCIH R00AT010903 (AP).

## Conflict of interest

The authors declare that the research was conducted in the absence of any commercial or financial relationships that could be construed as a potential conflict of interest.

## Publisher’s note

All claims expressed in this article are solely those of the authors and do not necessarily represent those of their affiliated organizations, or those of the publisher, the editors and the reviewers. Any product that may be evaluated in this article, or claim that may be made by its manufacturer, is not guaranteed or endorsed by the publisher.

## References

[ref1] Albrecht A. Stork O. (2017). Circadian rhythms in fear conditioning: an overview of behavioral, brain system, and molecular interactions. Neural Plast. 2017, 1–12. doi: 10.1155/2017/3750307, PMID: 28698810 PMC5494081

[ref2] Albrecht A. Thiere M. Bergado-Acosta J. R. Poranzke J. Müller B. Stork O. (2013). Circadian modulation of anxiety: a role for somatostatin in the amygdala. PLoS One 8, 1–9. doi: 10.1371/journal.pone.0084668 PMC3869835 24376834

[ref3] Alkozei A. Dailey N. S. Bajaj S. Vanuk J. R. Raikes A. C. Killgore W. D. S. (2021). Exposure to blue wavelength light is associated with increases in bidirectional amygdala-DLPFC connectivity at rest. Front. Neurol. 12, 1–11. doi: 10.3389/fneur.2021.625443 PMC8032953 33841300

[ref4] An K. Zhao H. Miao Y. Qi X. Li Y. F. Ma Y. Q.. (2020). A circadian rhythm-gated subcortical pathway for nighttime-light-induced depressive-like behaviors in mice. Nat. Neurosci. 23, 869–880. doi: 10.1038/s41593-020-0640-8, PMID: 32483349

[ref5] Andreatta G. Allen C. N. (2021). Circadian rhythm: how neurons adjust to diurnality. eLife 10:e74704. doi: 10.7554/eLife.74704 34845985 PMC8631939

[ref6] Arborelius L. Owens M. J. Plotsky P. M. Nemeroff C. B. (1999). The role of corticotropin-releasing factor in depression and anxiety disorders. J. Endocrinol. 160, 1–12. doi: 10.1677/joe.0.1600001, PMID: 9854171

[ref7] Arey R. N. Enwright J. F. Spencer S. M. Falcon E. Ozburn A. R. Ghose S.. (2014). An important role for cholecystokinin, a CLOCK target gene, in the development and treatment of manic-like behaviors. Mol. Psychiatry 19, 342–350. doi: 10.1038/mp.2013.12, PMID: 23399917 PMC3783638

[ref8] Arushanyan É. B. Popov A. V. (1995). Influence of damage to the suprachiasmatic nuclei of the hypothalamus of rats on the dynamics of short-period fluctuations of normal and abnormal behavior. Neurosci. Behav. Physiol. 25, 290–295. doi: 10.1007/BF02360039, PMID: 8570034

[ref9] Astiz M. Heyde I. Oster H. (2019). Mechanisms of communication in the mammalian circadian timing system. Int. J. Mol. Sci. 20:343. doi: 10.3390/ijms20020343, PMID: 30650649 PMC6359556

[ref10] Bae K. Jin X. Maywood E. S. Hastings M. H. Reppert S. M. Weaver D. R. (2001). Differential functions of MPer1, MPer2, and MPer3 in the SCN circadian clock. Neuron 30, 525–536. doi: 10.1016/S0896-6273(01)00302-6, PMID: 11395012

[ref11] Becker-Krail D. D. Ketchesin K. D. Burns J. N. Zong W. Hildebrand M. A. DePoy L. M.. (2022a). Astrocyte molecular clock function in the nucleus accumbens is important for reward-related behavior. Biol. Psychiatry 92, 68–80. doi: 10.1016/j.biopsych.2022.02.007, PMID: 35461698 PMC9232937

[ref12] Becker-Krail D. D. Walker W. H. Nelson R. J. (2022b). The ventral tegmental area and nucleus accumbens as circadian oscillators: implications for drug abuse and substance use disorders. Front. Physiol. 13, 1–17. doi: 10.3389/fphys.2022.886704 PMC9094703 35574492

[ref13] Bell-McGinty S. Butters M. A. Meltzer C. C. Greer P. J. Reynolds C. F. Becker J. T. (2002). Brain morphometric abnormalities in geriatric depression: long-term neurobiological effects of illness duration. Am. J. Psychiatr. 159, 1424–1427. doi: 10.1176/appi.ajp.159.8.1424, PMID: 12153839

[ref14] Ben-Hamo M. Larson T. A. Duge L. S. Sikkema C. Wilkinson C. W. de la Iglesia H. O.. (2017). Circadian forced desynchrony of the master clock leads to phenotypic manifestation of depression in rats. eNeuro. 3:ENEURO.0237–16.2016. doi: 10.1523/ENEURO.0237-16.2016 PMC5216685 28090585

[ref15] Blume C. Garbazza C. Spitschan M. (2019). Effects of light on human circadian rhythms, sleep and mood. Somnologie 23, 147–156. doi: 10.1007/s11818-019-00215-x, PMID: 31534436 PMC6751071

[ref16] Boehler N. A. Fung S. W. Hegazi S. Cheng A. H. Cheng H. Y. M. (2021). Sox2 ablation in the suprachiasmatic nucleus perturbs anxiety-and depressive-like behaviors. Neurol. Int. 13, 541–554. doi: 10.3390/neurolint13040054, PMID: 34842772 PMC8628992

[ref17] Bowden J. B. Abraham W. C. Harris K. M. (2012). Differential effects of strain, circadian cycle, and stimulation pattern on LTP and concurrent LTD in the dentate gyrus of freely moving rats. Hippocampus 22, 1363–1370. doi: 10.1002/hipo.20972, PMID: 21853503 PMC3292688

[ref18] Brancaccio M. Patton A. P. Chesham J. E. Maywood E. S. Hastings M. H. (2017). Astrocytes control circadian timekeeping in the suprachiasmatic nucleus via glutamatergic signaling. Neuron 93, 1420–1435.e5. doi: 10.1016/j.neuron.2017.02.030, PMID: 28285822 PMC5376383

[ref19] Castañeda T. R. De Prado B. M. Prieto D. Mora F. (2004). Circadian rhythms of dopamine, glutamate and GABA in the striatum and nucleus accumbens of the awake rat: modulation by light. J. Pineal Res. 36, 177–185. doi: 10.1046/j.1600-079X.2003.00114.x, PMID: 15009508

[ref20] Chaudhury D. Wang L. M. Colwell C. S. (2005). Circadian regulation of hippocampal long-term potentiation. J. Biol. Rhythm. 20, 225–236. doi: 10.1177/0748730405276352, PMID: 15851529 PMC2581477

[ref21] Chellappa S. L. Gaggioni G. Ly J. Q. M. Papachilleos S. Borsu C. Brzozowski A.. (2016). Circadian dynamics in measures of cortical excitation and inhibition balance. Sci. Rep. 6, 1–13. doi: 10.1038/srep33661 27651114 PMC5030482

[ref22] Cheng A. H. Bouchard-Cannon P. Hegazi S. Lowden C. Fung S. W. Chiang C. K.. (2019). SOX2-dependent transcription in clock neurons promotes the robustness of the central circadian pacemaker. Cell Rep. 26, 3191–3202.e8. doi: 10.1016/j.celrep.2019.02.068, PMID: 30893593

[ref23] Cheng J. Huang X. Liang Y. Xue T. Wang L. Bao J. (2018). Plasticity of light-induced concurrent glutamatergic and GABAergic quantal events in the suprachiasmatic nucleus. J. Biol. Rhythm. 33, 65–75. doi: 10.1177/0748730417754162 29432701

[ref24] Chung S. Lee E. J. Yun S. Choe H. K. Park S. B. Son H. J.. (2014). Impact of circadian nuclear receptor REV-ERBα on midbrain dopamine production and mood regulation. Cells 157, 858–868. doi: 10.1016/j.cell.2014.03.039, PMID: 24813609

[ref25] Ciarleglio C. M. Resuehr H. E. S. McMahon D. G. (2011). Interactions of the serotonin and circadian systems: nature and nurture in rhythms and blues. Neuroscience 197, 8–16. doi: 10.1016/j.neuroscience.2011.09.036, PMID: 21963350

[ref26] Coque L. Mukherjee S. Cao J. L. Spencer S. Marvin M. Falcon E.. (2011). Specific role of VTA dopamine neuronal firing rates and morphology in the reversal of anxiety-related, but not depression-related behavior in the clockδ19 mouse model of mania. Neuropsychopharmacology 36, 1478–1488. doi: 10.1038/npp.2011.33, PMID: 21430648 PMC3096816

[ref27] De Bundel D. Gangarossa G. Biever A. Bonnefont X. Valjent E. (2013). Cognitive dysfunction, elevated anxiety, and reduced cocaine response in circadian clock-deficient cryptochrome knockout mice. Front. Behav. Neurosci. 24:152. doi: 10.3389/fnbeh.2013.00152 PMC3807562 24187535

[ref28] DePoy L. M. McClung C. A. Logan R. W. (2017). Neural mechanisms of circadian regulation of natural and drug reward. Neural Plast.:5720842. doi: 10.1155/2017/5720842, PMID: 29359051 PMC5735684

[ref29] Domínguez-López S. Howell R. D. López-Canúl M. G. Leyton M. Gobbi G. (2014). Electrophysiological characterization of dopamine neuronal activity in the ventral tegmental area across the light-dark cycle. Synapse 68, 454–467. doi: 10.1002/syn.21757, PMID: 24955825

[ref30] Dulcis D. Pouya J. Leutgeb S. Spitzer N. C. (2013). Neurotransmitter switching in the adult brain regulates behavior. Science 40, 449–453. doi: 10.1146/annurev-neuro-072116-031204 23620046

[ref31] Duman R. S. Monteggia L. M. (2006). A neurotrophic model for stress-related mood disorders. Biol. Psychiatry 59, 1116–1127. doi: 10.1016/j.biopsych.2006.02.013, PMID: 16631126

[ref32] Dzirasa K. Coque L. Sidor M. M. Kumar S. Dancy E. A. Takahashi J. S.. (2010). Lithium Ameliorates nucleus accumbens phase-signaling dysfunction in a genetic mouse model of mania. J. Neurosci. 30, 16314–16323. doi: 10.1523/JNEUROSCI.4289-10.2010, PMID: 21123577 PMC3165036

[ref33] Easton A. Arbuzova J. Turek F. W. (2003). The circadian clock mutation increases exploratory activity and escape-seeking behavior. Genes Brain Behav. 2, 11–19. doi: 10.1034/j.1601-183X.2003.00002.x, PMID: 12882315

[ref34] Engin E. Stellbrink J. Treit D. Dickson C. T. (2008). Anxiolytic and antidepressant effects of intracerebroventricularly administered somatostatin: behavioral and neurophysiological evidence. Neuroscience 157, 666–676. doi: 10.1016/j.neuroscience.2008.09.037, PMID: 18940236

[ref35] Etain B. Milhiet V. Bellivier F. Leboyer M. (2011). Genetics of circadian rhythms and mood spectrum disorders. Eur. Neuropsychopharmacol. 21, S676–S682. doi: 10.1016/j.euroneuro.2011.07.007 21835597

[ref36] Evans J. A. Leise T. L. Castanon-Cervantes O. Davidson A. J. (2013). Dynamic interactions mediated by nonredundant signaling mechanisms couple circadian clock neurons. Neuron 80, 973–983. doi: 10.1016/j.neuron.2013.08.022, PMID: 24267653 PMC3841113

[ref37] Fernandez D. C. Chang Y.-T. Hattar S. Chen S.-K. (2016). Architecture of retinal projections to the central circadian pacemaker. Proc. Natl. Acad. Sci. 113, 6047–6052. doi: 10.1073/pnas.1523629113, PMID: 27162356 PMC4889372

[ref38] Fernandez D. C. Michelle Fogerson P. Ospri L. L. Thomsen M. B. Layne R. M. Severin D.. (2018). Light affects mood and learning through distinct retina-brain pathways. Cells 175, P71-84.E18. doi: 10.1016/j.cell.2018.08.004 PMC6190605 30173913

[ref39] Ferris M. J. España R. A. Locke J. L. Konstantopoulos J. K. Rose J. H. Chen R.. (2014). Dopamine transporters govern diurnal variation in extracellular dopamine tone. Proc. Natl. Acad. Sci. U. S. A. 111, 2751–2759. doi: 10.1073/pnas.1407935111 PMC4084435 24979798

[ref40] Fonken L. K. Aubrecht T. G. Hecmarie Meléndez-Fernández O. Weil Z. M. Nelson R. J. (2013). Dim light at night disrupts molecular circadian rhythms and increases body weight. J. Biol. Rhythm. 28, 262–271. doi: 10.1177/0748730413493862, PMID: 23929553 PMC4033305

[ref41] Fonken L. K. Kitsmiller E. Smale L. Nelson R. J. (2012). Dim Nighttime light impairs cognition and provokes depressive-like responses in a diurnal rodent. J. Biol. Rhythm. 27, 319–327. doi: 10.1177/0748730412448324, PMID: 22855576

[ref42] Fonken L. K. Sima Finy M. Walton J. C. Weil Z. M. Workman J. L. Ross J.. (2009). Influence of light at night on murine anxiety- and depressive-like responses. Behav. Brain Res. 205, 349–354. doi: 10.1016/j.bbr.2009.07.001, PMID: 19591880

[ref43] Fox M. E. Lobo M. K. (2019). The molecular and cellular mechanisms of depression: a focus on reward circuitry. Mol. Psychiatry 24, 1798–1815. doi: 10.1038/s41380-019-0415-3, PMID: 30967681 PMC6785351

[ref44] Francis T. C. Chandra R. Friend D. M. Finkel E. Dayrit G. Miranda J.. (2015). Nucleus accumbens medium spiny neuron subtypes mediate depression-related outcomes to social defeat stress. Biol. Psychiatry 77, 212–222. doi: 10.1016/j.biopsych.2014.07.021, PMID: 25173629 PMC5534173

[ref45] Francis T. C. Lobo M. K. (2017). Emerging role for nucleus accumbens medium spiny neuron subtypes in depression. Biol. Psychiatry 81, 645–653. doi: 10.1016/j.biopsych.2016.09.007, PMID: 27871668 PMC5352537

[ref46] Francis T. C. Yano H. Demarest T. G. Shen H. Bonci A. (2019). High-frequency activation of nucleus accumbens D1-MSNs drives excitatory potentiation on D2-MSNs. Neuron 103, 432–444.e3. doi: 10.1016/j.neuron.2019.05.031, PMID: 31221559 PMC6712577

[ref47] Garbazza C. Benedetti F. (2018). Genetic factors affecting seasonality, mood, and the circadian clock. Front. Endocrinol. 23:481. doi: 10.3389/fendo.2018.00481 PMC6115502 30190706

[ref48] Garcia J. A. Zhang D. Estill S. J. Michnoff C. Rutter J. Reick M.. (2000). Impaired cued and contextual memory in NPAS2-deficient mice. Science 288, 2226–2230. 10864874 10.1126/science.288.5474.2226

[ref49] Gerlach A. R. Karim H. T. Peciña M. Ajilore O. Taylor W. D. Butters M. A.. (2022). MRI predictors of pharmacotherapy response in major depressive disorder. NeuroImage 36:103157. doi: 10.1016/j.nicl.2022.103157 36027717 PMC9420953

[ref50] Glass J. D. Grossman G. H. Farnbauch L. DiNardo L. (2003). Midbrain raphe modulation of nonphotic circadian clock resetting and 5-HT release in the mammalian suprachiasmatic nucleus. J. Neurosci. 23, 7451–7460. doi: 10.1523/JNEUROSCI.23-20-07451.2003, PMID: 12930783 PMC6740771

[ref51] Golombek D. A. Rosenstein R. E. (2010). Physiology of circadian entrainment. Physiol. Rev. 90, 1063–1102. doi: 10.1152/physrev.00009.2009, PMID: 20664079

[ref52] Gonzalez J. C. Lee H. Vincent A. M. Gamble K. L. Wadiche J. I. Overstreet-wadiche L.. (2023). Article circadian regulation of dentate gyrus excitability mediated by G-protein signaling Ll Ll circadian regulation of dentate gyrus excitability mediated by G-protein signaling. CellReports 42:112039. doi: 10.1016/j.celrep.2023.112039 PMC10404305 36749664

[ref53] Goriki A. Hatanaka F. Myung J. Kim J. K. Yoritaka T. Tanoue S.. (2014). A novel protein, CHRONO, functions as a core component of the mammalian circadian clock. PLoS Biol. 12:e1001839. doi: 10.1371/journal.pbio.1001839, PMID: 24736997 PMC3988004

[ref54] Güler A. D. Ecker J. L. Lall G. S. Haq S. Altimus C. M. Liao H. W.. (2008). Melanopsin cells are the principal conduits for Rod-cone input to non-image-forming vision. Nature 453, 102–105. doi: 10.1038/nature06829, PMID: 18432195 PMC2871301

[ref55] Hallahan B. Newell J. Soares J. C. Brambilla P. Strakowski S. M. Fleck D. E.. (2011). Structural magnetic resonance imaging in bipolar disorder: an international collaborative mega-analysis of individual adult patient data. Biol. Psychiatry 69, 326–335. doi: 10.1016/j.biopsych.2010.08.029, PMID: 21030008

[ref56] Hamo M. B. Larson T. A. Duge L. S. Sikkema C. Wilkinson C. W. De La Iglesia H. O.. (2016). Circadian forced desynchrony of the master clock leads to phenotypic manifestation of depression in rats. ENeuro 3, 1–13. doi: 10.1523/ENEURO.0237-16.2016 PMC5216685 28090585

[ref57] Hampp G. Ripperger J. A. Houben T. Schmutz I. Blex C. Perreau-Lenz S.. (2008). Regulation of monoamine oxidase a by circadian-clock components implies clock influence on mood. Curr. Biol. 18, 678–683. doi: 10.1016/j.cub.2008.04.012, PMID: 18439826

[ref58] Harris K. M. Teyler T. J. (1983). Age differences in a circadian influence on hippocampal LTP. Brain Res. 261, 69–73. doi: 10.1016/0006-8993(83)91284-2, PMID: 6301629

[ref59] Hattar S. Kumar M. Park A. Tong P. (2006). Central projections of melanopsin-expressing retinal ganglion cells in the mouse. J. Comp. Neurol. 497, 326–349. doi: 10.1002/cne.20970, PMID: 16736474 PMC2885916

[ref60] Hensel L. Rohleder N. Niessen C. (2022). Effects of psychosocial stress on prosociality: the moderating role of current life stress and thought control. Stress 25, 235–245. doi: 10.1080/10253890.2022.2054697, PMID: 35713555

[ref61] Honoré E. Khlaifia A. Bosson A. Lacaille J. C. (2021). Hippocampal somatostatin interneurons, long-term synaptic plasticity and memory. Front. Neural. Circuit. 15, 1–24. doi: 10.3389/fncir.2021.687558 PMC8206813 34149368

[ref62] Huang L. Xi Y. Peng Y. Yang Y. Huang X. Yunwei F.. (2019). A visual circuit related to habenula underlies the antidepressive effects of light therapy. Neuron 102, 128–142.e8. doi: 10.1016/j.neuron.2019.01.037, PMID: 30795900

[ref63] Hühne A. Volkmann P. Stephan M. Rossner M. Landgraf D. (2020). An in-depth neurobehavioral characterization shows anxiety-like traits, impaired habituation behavior, and restlessness in male cryptochrome-deficient mice. Genes Brain Behav. 19, 1–15. doi: 10.1111/gbb.12661 32348614

[ref64] Imamura K. Takumi T. (2022). Mood phenotypes in rodent models with circadian disturbances. Neurobiol. Sleep Circad. Rhythms 13:100083. doi: 10.1016/j.nbscr.2022.100083 PMC9636574 36345502

[ref65] Jilg A. Lesny S. Peruzki N. Schwegler H. Selbach O. Dehghani F.. (2010). Temporal dynamics of mouse hippocampal clock gene expression support memory processing. Hippocampus 20, 377–388. doi: 10.1002/hipo.20637, PMID: 19437502

[ref66] Jones J. R. Chaturvedi S. Granados-Fuentes D. Herzog E. D. (2021). Circadian neurons in the paraventricular nucleus entrain and sustain daily rhythms in glucocorticoids. Nat. Commun. 12, 1–15. doi: 10.1038/s41467-021-25959-9 34599158 PMC8486846

[ref67] Kawai H. Iwadate R. Ishibashi T. Kudo N. Kawashima Y. Mitsumoto A. (2019). Antidepressants with different mechanisms of action show different chronopharmacological profiles in the tail suspension test in mice. Chronobiol. Int. 36, 1194–1207. doi: 10.1080/07420528.2019.1625360, PMID: 31198056

[ref68] Kim R. Healey K. L. Sepulveda-Orengo M. T. Reissner K. J. (2018). Astroglial correlates of neuropsychiatric disease: from astrocytopathy to astrogliosis. Prog. Neuro Psychopharmacol. Biol. Psychiatry 87, 126–146. doi: 10.1016/j.pnpbp.2017.10.002 PMC5889368 28989099

[ref69] Kripke D. F. Nievergelt C. M. Joo E. J. Shekhtman T. Kelsoe J. R. (2009). Circadian polymorphisms associated with affective disorders. J. Circadian Rhythms 7, 1–10. doi: 10.1186/1740-3391-7-2 19166596 PMC2661876

[ref70] Kwak Y. Jeong J. Lee S. Park Y. U. Lee S. A. Han D. H.. (2013). Cyclin-dependent kinase 5 (Cdk5) regulates the function of CLOCK protein by direct phosphorylation. J. Biol. Chem. 288, 36878–36889. doi: 10.1074/jbc.M113.494856, PMID: 24235147 PMC3873547

[ref71] Lamont E. W. Robinson B. Stewart J. Amir S. (2005). The central and basolateral nuclei of the amygdala exhibit opposite diurnal rhythms of expression of the clock protein Period2. Proc. Natl. Acad. Sci. U. S. A. 102, 4180–4184. doi: 10.1073/pnas.0500901102, PMID: 15746242 PMC554834

[ref72] Landgraf D. Long J. Der-Avakian A. Streets M. Welsh D. K. (2015). Dissociation of learned helplessness and fear conditioning in mice: a mouse model of depression. PLoS One 10, 1–17. doi: 10.1371/journal.pone.0125892 PMC4416012 25928892

[ref73] Landgraf D. Long J. E. Proulx C. D. Barandas R. Malinow R. Welsh D. K. (2016a). Genetic disruption of circadian rhythms in the suprachiasmatic nucleus causes helplessness, behavioral despair, and anxiety-like behavior in mice. Biol. Psychiatry 80, 827–835. doi: 10.1016/j.biopsych.2016.03.1050, PMID: 27113500 PMC5102810

[ref74] Landgraf D. Long J. E. Welsh D. K. (2016b). Depression-like behaviour in mice is associated with disrupted circadian rhythms in nucleus accumbens and periaqueductal grey. Eur. J. Neurosci. 43, 1309–1320. doi: 10.1111/ejn.13085, PMID: 26414405

[ref75] Lee I. T. Chang A. S. Manandhar M. Shan Y. Fan J. Izumo M.. (2015). Neuromedin S-producing neurons act as essential pacemakers in the suprachiasmatic nucleus to couple clock neurons and dictate circadian rhythms. Neuron 85, 1086–1102. doi: 10.1016/j.neuron.2015.02.006, PMID: 25741729 PMC5811223

[ref76] Legates T. A. Fernandez D. C. Hattar S. (2014). Light as a central modulator of circadian rhythms, sleep and affect. Nat. Rev. Neurosci. 15, 443–454. doi: 10.1038/nrn3743, PMID: 24917305 PMC4254760

[ref77] LeGates T. A. Kvarta M. D. Tooley J. R. Chase Francis T. Lobo M. K. Creed M. C.. (2018). Reward behaviour is regulated by the strength of hippocampus–nucleus accumbens synapses. Nature 564, 258–262. doi: 10.1038/s41586-018-0740-8, PMID: 30478293 PMC6292781

[ref78] Li J. Z. Bunney B. G. Meng F. Hagenauer M. H. Walsh D. M. Vawter M. P.. (2013). Circadian patterns of gene expression in the human brain and disruption in major depressive disorder. Proc. Natl. Acad. Sci. U. S. A. 110, 9950–9955. doi: 10.1073/pnas.1305814110, PMID: 23671070 PMC3683716

[ref79] Li Y. Li G. Li J. Cai X. Sun Y. Zhang B.. (2021). Depression-like behavior is associated with lower Per2 MRNA expression in the lateral habenula of rats. Genes Brain Behav. 20, 1–10. doi: 10.1111/gbb.12702 32964673

[ref80] Li K. Zhou T. Liao L. Yang Z. Wong C. Henn F.. (2013). ΒCaMKII in lateral habenula mediates core symptoms of depression. Science 341, 1016–1020. doi: 10.1126/science.1240729, PMID: 23990563 PMC3932364

[ref81] Lim B. K. Huang K. W. Grueter B. A. Rothwell P. E. Malenka R. C. (2012). Anhedonia requires MC4R-mediated synaptic adaptations in nucleus accumbens. Nature 487, 183–189. doi: 10.1038/nature11160, PMID: 22785313 PMC3397405

[ref82] Lobo M. K. Covington III H. E. Chaudhury D. Friedman A. K. Sun H. S. Damez-Werno D.. (2010). Cell type–specific loss of BDNF signaling mimics optogenetic control of cocaine reward. Science 330, 385–390. doi: 10.1126/science.1188472 20947769 PMC3011229

[ref83] Lobo M. K. Nestler E. J. (2011). The striatal balancing act in drug addiction: distinct roles of direct and indirect pathway medium spiny neurons. Front. Neuroanat. 5, 1–11. doi: 10.3389/fnana.2011.00041 21811439 PMC3140647

[ref84] Logan R. W. Edgar N. Gillman A. G. Hoffman D. Zhu X. McClung C. A. (2015). Chronic stress induces brain region-specific alterations of molecular rhythms that correlate with depression-like behavior in mice. Biol. Psychiatry 78, 249–258. doi: 10.1016/j.biopsych.2015.01.011 25771506 PMC4509914

[ref85] Logan R. W. McClung C. A. (2018). Rhythms of life: circadian disruption and brain disorders across the lifespan. Nat. Rev. Neurosci. 49–65. doi: 10.1038/s41583-018-0088-y 30459365 PMC6338075

[ref86] Logan R. W. Parekh P. K. Kaplan G. N. Becker-Krail D. D. Williams W. P. Yamaguchi S.. (2018). NAD+ cellular redox and SIRT1 regulate the diurnal rhythms of tyrosine hydroxylase and conditioned cocaine reward. Mol. Psychiatry 24, 1668–1684. doi: 10.1038/s41380-018-0061-1 29728703 PMC6215755

[ref87] Malek Z. S. Dardente H. Pevet P. Raison S. (2005). Tissue-specific expression of tryptophan hydroxylase MRNAs in the rat midbrain: anatomical evidence and daily profiles. Eur. J. Neurosci. 22, 895–901. doi: 10.1111/j.1460-9568.2005.04264.x, PMID: 16115212

[ref88] Malek Z. S. Sage D. Pévet P. Raison S. (2007). Daily rhythm of tryptophan hydroxylase-2 messenger ribonucleic acid within raphe neurons is induced by corticoid daily surge and modulated by enhanced locomotor activity. Endocrinology 148, 5165–5172. doi: 10.1210/en.2007-0526, PMID: 17595225

[ref89] Martini T. Ripperger J. A. Stalin J. Kores A. Stumpe M. Albrecht U. (2021). Deletion of the clock gene Period2 (Per2) in glial cells alters mood-related behavior in mice. Sci. Rep. 11, 1–15. doi: 10.1038/s41598-021-91770-7 34112905 PMC8192521

[ref90] McCarthy M. J. Wei H. Marnoy Z. Darvish R. M. McPhie D. L. Cohen B. M.. (2013). Genetic and clinical factors predict lithium’s effects on PER2 gene expression rhythms in cells from bipolar disorder patients. Transl. Psychiatry 3:e318. doi: 10.1038/tp.2013.90, PMID: 24150227 PMC3818008

[ref91] McCarthy M. J. Welsh D. K. (2012). Cellular circadian clocks in mood disorders. J. Biol. Rhythm. 27, 339–352. doi: 10.1177/0748730412456367, PMID: 23010657

[ref92] McCauley J. P. Petroccione M. A. D’Brant L. Y. Todd G. C. Affinnih N. Wisnoski J. J.. (2020). Circadian modulation of neurons and astrocytes controls synaptic plasticity in hippocampal area CA1. Cell Rep. 33:108255. doi: 10.1016/j.celrep.2020.108255, PMID: 33053337 PMC7700820

[ref93] McClung C. A. (2007). Circadian genes, rhythms and the biology of mood disorders. Pharmacol. Ther. 114, 222–232. doi: 10.1016/j.pharmthera.2007.02.003, PMID: 17395264 PMC1925042

[ref94] McClung C. A. Sidiropoulou K. Vitaterna M. Takahashi J. S. White F. J. Cooper D. C.. (2005). Regulation of dopaminergic transmission and cocaine reward by the clock gene. Proc. Natl. Acad. Sci. U. S. A. 102, 9377–9381. doi: 10.1073/pnas.0503584102, PMID: 15967985 PMC1166621

[ref95] McDonald A. J. Mascagni F. Augustine J. R. (1995). Neuropeptide Y and somatostatin-like immunoreactivity in neurons of the monkey amygdala. Neuroscience 66, 959–982. doi: 10.1016/0306-4522(94)00629-J, PMID: 7651623

[ref96] Milosavljevic N. Cehajic-Kapetanovic J. Procyk C. A. Lucas R. J. (2016). Chemogenetic Activation of Melanopsin Retinal Ganglion Cells Induces Signatures of Arousal and/or Anxiety in Mice. Curr. Biol. 26, 2358–2363. doi: 10.1016/j.cub.2016.06.057, PMID: 27426512 PMC5026697

[ref97] Moore R. Y. Speh J. C. Leak R. K. (2002). Suprachiasmatic nucleus organization. Cell Tissue Res. 309, 89–98. doi: 10.1007/s00441-002-0575-2, PMID: 12111539

[ref98] Mori K. Miyazato M. Ida T. Murakami N. Serino R. Ueta Y.. (2005). Identification of neuromedin S and its possible role in the mammalian circadian oscillator system. EMBO J. 24, 325–335. doi: 10.1038/sj.emboj.7600526, PMID: 15635449 PMC545815

[ref99] Morin L. P. Meyer-Bernstein E. L. (1999). Electrical stimulation of the median or dorsal raphe nuclei reduces light-induced FOS protein in the suprachiasmatic nucleus and causes circadian activity rhythm phase shifts. Neuroscience 92, 267–279. 10392849 10.1016/s0306-4522(98)00733-7

[ref100] Mukherjee S. Coque L. Cao J. L. Kumar J. Chakravarty S. Asaithamby A.. (2010). Knockdown of clock in the ventral tegmental Area through RNA interference results in a mixed state of mania and depression-like behavior. Biol. Psychiatry 68, 503–511. doi: 10.1016/j.biopsych.2010.04.031, PMID: 20591414 PMC2929276

[ref101] Nestler E. J. (2015). Role of the brain’s reward circuitry in depression: transcriptional mechanisms. Int. Rev. Neurobiol 124, 151–170. doi: 10.1016/bs.irn.2015.07.003 26472529 PMC4690450

[ref102] Olejniczak I. Ripperger J. A. Sandrelli F. Schnell A. Mansencal-Strittmatter L. Wendrich K.. (2021). Light affects behavioral despair involving the clock gene period 1. PLoS Genet. 17, 1–24. doi: 10.1371/journal.pgen.1009625 PMC8266116 34237069

[ref103] Ono D. Honma K. I. Honma S. (2021). Roles of neuropeptides, VIP and AVP, in the mammalian central circadian clock. Front. Neurosci. 15, 1–8. doi: 10.3389/fnins.2021.650154 PMC8081951 33935635

[ref104] Ozburn A. R. Falcon E. Mukherjee S. Gillman A. Arey R. Spencer S.. (2013). The role of clock in ethanol-related behaviors. Neuropsychopharmacology 38, 2393–2400. doi: 10.1038/npp.2013.138, PMID: 23722243 PMC3799058

[ref105] Ozburn A. R. Kern J. Parekh P. K. Logan R. W. Liu Z. Falcon E.. (2017). NPAS2 regulation of anxiety-like behavior and GABAA receptors. Front. Mol. Neurosci. 10, 1–12. doi: 10.3389/fnmol.2017.00360 29163035 PMC5675889

[ref106] Pantazopoulos H. Wiseman J. T. Markota M. Ehrenfeld L. Berretta S. (2017). Decreased numbers of somatostatin-expressing neurons in the amygdala of subjects with bipolar disorder or schizophrenia: relationship to circadian rhythms. Biol. Psychiatry 81, 536–547. doi: 10.1016/j.biopsych.2016.04.006, PMID: 27259817 PMC5065936

[ref107] Parekh P. K. Becker-Krail D. Sundaravelu P. Ishigaki S. Okado H. Sobue G.. (2017). Altered GluA1 (Gria1) function and accumbal synaptic plasticity in the Clockδ19 model of bipolar mania. Biol. Psychiatry 84, 817–826. doi: 10.1016/j.biopsych.2017.06.022 28780133 PMC5745309

[ref108] Parekh P. K. Logan R. W. Ketchesin K. D. Becker-Krail D. Shelton M. A. Hildebrand M. A.. (2019). Cell-type-specific regulation of nucleus accumbens synaptic plasticity and cocaine reward sensitivity by the circadian protein, NPAS2. J. Neurosci. 39, 4657–4667. doi: 10.1523/JNEUROSCI.2233-18.2019, PMID: 30962277 PMC6561687

[ref109] Parekh P. K. McClung C. A. (2016). Circadian mechanisms underlying reward-related neurophysiology and synaptic plasticity. Front. Psych. 6, 1–11. doi: 10.3389/fpsyt.2015.00187 PMC4709415 26793129

[ref110] Paul J. R. Davis J. A. Goode L. K. Becker B. K. Fusilier A. Meador-Woodruff A.. (2020). Circadian regulation of membrane physiology in neural oscillators throughout the brain. Eur. J. Neurosci. 51, 109–138. doi: 10.1111/ejn.14343, PMID: 30633846 PMC6625955

[ref111] Pittenger C. Duman R. S. (2008). Stress, depression, and neuroplasticity: a convergence of mechanisms. Neuropsychopharmacology 33, 88–109. doi: 10.1038/sj.npp.1301574, PMID: 17851537

[ref112] Porcu A. Nilsson A. Booreddy S. Barnes S. A. Welsh D. K. Dulcis D. (2022). Seasonal changes in day length induce multisynaptic neurotransmitter switching to regulate hypothalamic network activity and behavior. Sci. Adv. 8:eabn9867. doi: 10.1126/sciadv.abn9867, PMID: 36054362 PMC10848959

[ref113] Porcu A. Vaughan M. Nilsson A. Arimoto N. Lamia K. Welsh D. K. (2020). Vulnerability to helpless behavior is regulated by the circadian clock component CRYPTOCHROME in the mouse nucleus accumbens. Proc. Natl. Acad. Sci. U. S. A. 117, 13771–13782. doi: 10.1073/pnas.2000258117, PMID: 32487727 PMC7306774

[ref114] Preitner N. Damiola F. Lopez-Molina L. Zakany J. Duboule D. Albrecht U.. (2002). Erratum: The orphan nuclear receptor REV-ERBα controls circadian transcription within the positive limb of the mammalian circadian oscillator (Cell 110 (251-260)). Cells 110:535. doi: 10.1016/S0092-8674(02)00898-X 12150932

[ref115] Prolo L. M. Takahashi J. S. Herzog E. D. (2005). Circadian rhythm generation and entrainment in astrocytes. J. Neurosci. 25, 404–408. doi: 10.1523/JNEUROSCI.4133-04.2005, PMID: 15647483 PMC3812245

[ref116] Pyter L. M. Nelson R. J. (2006). Enduring effects of photoperiod on affective behaviors in Siberian hamsters (*Phodopus Sungorus*). Behav. Neurosci. 120, 125–134. doi: 10.1037/0735-7044.120.1.125, PMID: 16492123

[ref117] Quina L. A. Tempest L. Ng L. Harris J. A. Ferguson S. Jhou T. C.. (2015). Efferent pathways of the mouse lateral habenula. J. Comp. Neurol. 523, 32–60. doi: 10.1002/cne.23662, PMID: 25099741 PMC4232452

[ref118] Ren C. Luan L. Lau B. W.-M. Huang X. Yang J. Zhou Y.. (2013). Direct retino-raphe projection alters serotonergic tone and affective behavior. Neuropsychopharmacology 38, 1163–1175. doi: 10.1038/npp.2013.35, PMID: 23370156 PMC3656380

[ref119] Romanov R. A. Zeisel A. Bakker J. Girach F. Hellysaz A. Tomer R.. (2017). Molecular interrogation of hypothalamic organization reveals distinct dopamine neuronal subtypes. Nat. Neurosci. 20, 176–188. doi: 10.1038/nn.4462, PMID: 27991900 PMC7615022

[ref120] Romijn H. J. Sluiter A. A. Pool C. W. Wortel J. Buijs R. M. (1997). Evidence from confocal fluorescence microscopy for a dense, reciprocal innervation between AVP-, somatostatin-, VIP/PHI-, GRP- and VIP/PHI/GRP-immunoreactive neurons in the rat suprachiasmatic nucleus. Eur. J. Neurosci. 9, 2613–2623. doi: 10.1111/j.1460-9568.1997.tb01691.x, PMID: 9517467

[ref121] Rosenwasser A. M. Turek F. W. (2010). “Physiology of the mammalian circadian system” in Principles and Practice of Sleep Medicine. 5th ed. eds. Kryger M. H. Roth T. Dement W. C. (Elsevier Inc.), 390–401.

[ref122] Roybal K. Theobold D. Graham A. DiNieri J. A. Russo S. J. Krishnan V.. (2007). Mania-like behavior induced by disruption of CLOCK. Proc. Natl. Acad. Sci. U. S. A. 104, 6406–6411. doi: 10.1073/pnas.0609625104, PMID: 17379666 PMC1851061

[ref123] Rubinow D. R. (1986). Cerebrospinal fluid somatostatin and psychiatric illness. Biol. Psychiatry 21, 341–365. doi: 10.1016/0006-3223(86)90163-0, PMID: 2869790

[ref124] Russo S. J. Nestler E. J. (2013). The brain reward circuitry in mood disorders. Nat. Rev. Neurosci. 14, 609–625. doi: 10.1038/nrn3381, PMID: 23942470 PMC3867253

[ref125] Sahlem G. L. Kalivas B. Fox J. B. Lamb K. Roper A. Williams E. N.. (2014). Adjunctive triple chronotherapy (combined total sleep deprivation, sleep phase advance, and bright light therapy) rapidly improves mood and suicidality in suicidal depressed inpatients: an open label pilot study. J. Psychiatr. Res. 59, 101–107. doi: 10.1016/j.jpsychires.2014.08.015, PMID: 25231629 PMC4252537

[ref126] Sakhi K. Belle M. D. C. Gossan N. Delagrange P. Piggins H. D. (2014a). Daily variation in the electrophysiological activity of mouse medial habenula neurones. J. Physiol. 592, 587–603. doi: 10.1113/jphysiol.2013.263319 24247982 PMC3934703

[ref127] Sakhi K. Wegner S. Belle M. D. C. Howarth M. Delagrange P. Brown T. M.. (2014b). Intrinsic and extrinsic cues regulate the daily profile of mouse lateral habenula neuronal activity. J. Physiol. 592, 5025–5045. doi: 10.1113/jphysiol.2014.280065 25194046 PMC4259541

[ref128] Savalli G. Diao W. Berger S. Ronovsky M. Partonen T. Pollak D. D. (2015a). Anhedonic behavior in cryptochrome 2-deficient mice is paralleled by altered diurnal patterns of amygdala gene expression. Amino Acids 47, 1367–1377. doi: 10.1007/s00726-015-1968-3, PMID: 25820768 PMC4458264

[ref129] Savalli G. Diao W. Schulz S. Todtova K. Pollak D. D. (2015b). Diurnal oscillation of amygdala clock gene expression and loss of synchrony in a mouse model of depression. Int. J. Neuropsychopharmacol. 18, 1–11. doi: 10.1093/ijnp/pyu095 PMC4376549 25522426

[ref130] Scofield M. D. Kalivas P. W. (2014). Astrocytic dysfunction and addiction: consequences of impaired glutamate homeostasis. Neuroscientist 20, 610–622. doi: 10.1177/1073858413520347, PMID: 24496610 PMC4913887

[ref131] Severino G. Manchia M. Contu P. Squassina A. Lampus S. Ardau R.. (2009). Association study in a Sardinian sample between bipolar disorder and the nuclear receptor REV-ERBα gene, a critical component of the circadian clock system. Bipolar Disord. 11, 215–220. doi: 10.1111/j.1399-5618.2009.00667.x, PMID: 19267705

[ref132] Sokolowska E. Viitanen R. Misiewicz Z. Mennesson M. Saarnio S. Kulesskaya N.. (2021). The circadian gene cryptochrome 2 influences stress-induced brain activity and depressive-like behavior in mice. Genes Brain Behav. 20, 1–12. doi: 10.1111/gbb.12708 33070440

[ref133] Spencer S. Falcon E. Kumar J. Krishnan V. Mukherjee S. Birnbaum S. G.. (2013). Circadian genes period 1 and period 2 in the nucleus accumbens regulate anxiety-related behavior. Eur. J. Neurosci. 37, 242–250. doi: 10.1111/ejn.12010, PMID: 23039899 PMC3711746

[ref134] Sprouse J. Braselton J. Reynolds L. (2006). Fluoxetine modulates the circadian biological clock via phase advances of suprachiasmatic nucleus neuronal firing. Biol. Psychiatry 60, 896–899. doi: 10.1016/j.biopsych.2006.03.003, PMID: 16631132

[ref135] Stamatakis A. M. Stuber G. D. (2012). Activation of lateral habenula inputs to the ventral midbrain promotes behavioral avoidance. Nat. Neurosci. 15, 1105–1107. doi: 10.1038/nn.3145, PMID: 22729176 PMC3411914

[ref136] Stowe T. A. Pitts E. G. Leach A. C. Iacino M. C. Niere F. Graul B.. (2022). Diurnal rhythms in cholinergic modulation of rapid dopamine signals and associative learning in the striatum. Cell Rep. 39:110633. doi: 10.1016/j.celrep.2022.110633, PMID: 35385720 PMC9148619

[ref137] Takahashi J. S. Hong H. K. Ko C. H. McDearmon E. L. (2008). The genetics of mammalian circadian order and disorder: implications for physiology and disease. Nat. Rev. Genet. 9, 764–775. doi: 10.1038/nrg2430, PMID: 18802415 PMC3758473

[ref138] Tancredi S. Urbano T. Vinceti M. Filippini T. (2022). Artificial light at night and risk of mental disorders: a systematic review. Sci. Total Environ. 833:155185. doi: 10.1016/j.scitotenv.2022.155185 35417728

[ref139] Tataroǧlu Ö. Aksoy A. Yilmaz A. Canbeyli R. (2004). Effect of lesioning the suprachiasmatic nuclei on behavioral despair in rats. Brain Res. 1001, 118–124. doi: 10.1016/j.brainres.2003.11.063 14972660

[ref140] Thompson S. M. Kallarackal A. J. Kvarta M. D. Van Dyke A. M. LeGates T. A. Cai X. (2015). An excitatory synapse hypothesis of depression. Trends Neurosci. 38, 279–294. doi: 10.1016/j.tins.2015.03.003, PMID: 25887240 PMC4417609

[ref141] Vadnie C. A. McClung C. A. (2017). Circadian rhythm disturbances in mood disorders: insights into the role of the suprachiasmatic nucleus. Neural Plast. 2017:1504507. doi: 10.1155/2017/1504507, PMID: 29230328 PMC5694588

[ref142] Vadnie C. A. Petersen K. A. Eberhardt L. A. Hildebrand M. A. Cerwensky A. J. Zhang H.. (2022). The suprachiasmatic nucleus regulates anxiety-like behavior in mice. Front. Neurosci. 15, 1–17. doi: 10.3389/fnins.2021.765850 PMC8811036 35126036

[ref143] Valeri J. O’Donovan S. M. Wang W. Sinclair D. Bollavarapu R. Gisabella B.. (2022). Altered expression of somatostatin signaling molecules and clock genes in the hippocampus of subjects with substance use disorder. Front. Neurosci. 16, 1–22. doi: 10.3389/fnins.2022.903941 PMC9489843 36161151

[ref144] VanderLeest H. T. Houben T. Michel S. Deboer T. Albus H. Vansteensel M. J.. (2007). Seasonal encoding by the circadian pacemaker of the SCN. Curr. Biol. 17, 468–473. doi: 10.1016/j.cub.2007.01.048, PMID: 17320387

[ref145] Vandewalle G. Schwartz S. Grandjean D. Wuillaume C. Balteau E. Degueldre C.. (2010). Spectral quality of light modulates emotional brainresponses in humans. Proc. Natl. Acad. Sci. U. S. A. 107, 19549–19554. doi: 10.1073/pnas.1010180107, PMID: 20974959 PMC2984196

[ref146] Walton J. C. Haim A. Spieldenner J. M. Nelson R. J. (2012). Photoperiod alters fear responses and basolateral amygdala neuronal spine density in white-footed mice (*Peromyscus Leucopus*). Behav. Brain Res. 233, 345–350. doi: 10.1016/j.bbr.2012.05.033, PMID: 22652395 PMC3402626

[ref147] Yang Y. Cui Y. Sang K. Dong Y. Ni Z. Ma S.. (2018). Ketamine blocks bursting in the lateral habenula to rapidly relieve depression. Nature 554, 317–322. doi: 10.1038/nature25509, PMID: 29446381

[ref148] Yeung M. Treit D. (2012). The anxiolytic effects of somatostatin following intra-septal and intra-amygdalar microinfusions are reversed by the Selective Sst2 antagonist PRL2903. Pharmacol. Biochem. Behav. 101, 88–92. doi: 10.1016/j.pbb.2011.12.012, PMID: 22210489

[ref149] Zhang R. Volkow N. D. (2023). Seasonality of brain function: role in psychiatric disorders. Transl. Psychiatry 13:65. doi: 10.1038/s41398-023-02365-x 36813773 PMC9947162

[ref150] Zhao C. Gammie S. C. (2018). The circadian gene Nr1d1 in the mouse nucleus accumbens modulates sociability and anxiety-related behaviour. Eur. J. Neurosci. 48, 1924–1943. doi: 10.1111/ejn.14066, PMID: 30028550 PMC6113111

[ref151] Zhao H. Rusak B. (2005). Circadian firing-rate rhythms and light responses of rat habenular nucleus neurons in vivo and in vitro. Neuroscience 132, 519–528. doi: 10.1016/j.neuroscience.2005.01.012, PMID: 15802202

[ref152] Zhou J. N. Riemersma R. F. Unmehopa U. A. Hoogendijk W. J. G. Van Heerikhuize J. J. Hofman M. A.. (2001). Alterations in arginine vasopressin neurons in the suprachiasmatic nucleus in depression. Arch. Gen. Psychiatry 58, 655–662. doi: 10.1001/archpsyc.58.7.655, PMID: 11448372

[ref153] Zhu W. L. Shi H. S. Wang S. J. Chun Mei X. Jiang W. G. Wang X.. (2012). Increased Cdk5/P35 activity in the dentate gyrus mediates depressive-like behaviour in rats. Int. J. Neuropsychopharmacol. 15, 795–809. doi: 10.1017/S1461145711000915 21682945

